# A middle energy-bandwidth X-ray monochromator for high-flux synchrotron diffraction: revisiting asymmetrically cut silicon crystals

**DOI:** 10.1107/S1600577519003473

**Published:** 2019-04-17

**Authors:** Hiroo Tajiri, Hiroshi Yamazaki, Haruhiko Ohashi, Shunji Goto, Osami Sakata, Tetsuya Ishikawa

**Affiliations:** a Japan Synchrotron Radiation Research Institute, 1-1-1 Kouto, Sayo, Hyogo 679-5198, Japan; b National Institute for Materials Science, 1-1-1 Kouto, Sayo, Hyogo 679-5148, Japan; c RIKEN SPring-8 Center, 1-1-1 Kouto, Sayo, Hyogo 679-5148, Japan

**Keywords:** X-ray monochromators, double-crystal monochromators, asymmetric geometry, middle energy-bandwidth, silicon crystals, high-flux diffraction

## Abstract

To supply the growing demand for high photon flux in synchrotron science including surface diffraction, a middle energy-bandwidth monochromator covering the 10^−4^ to 10^−3^ range has been adapted by applying an asymmetric diffraction geometry to a cryogenically cooled silicon 111 double-crystal monochromator used as a standard for the undulator source at SPring-8. The asymmetric geometry provides a great advantage with its ability to configure flux gains over a wide energy range by simply changing the asymmetry angle, while the angular divergence of the exit beam remains unchanged. A monolithic design with three faces has been employed, having one symmetrically cut and another two asymmetrically cut surfaces relative to the same atomic plane, maintaining cooling efficiency and the capability of quickly changing the reflection surface. With the asymmetric geometry, an X-ray flux greater than 10^14^ photons s^−1^ was available around 12 keV. A maximum gain of 2.5 was obtained relative to the standard symmetric condition.

## Abstract 2

Using an asymmetric diffraction geometry, a silicon double-crystal monochromator can produce a higher photon flux than the standard symmetric geometry for low-emittance synchrotron radiation X-rays, while the outgoing beam from the monochromator maintains its narrow angular spread. These features are well suited to synchrotron diffraction and scattering.

## 1. Introduction   

Recent progress in synchrotron radiation sources, especially low-emittance undulator sources, allows the angular divergence of the X-ray beam from the undulator to become narrower than the acceptance width of a crystal monochromator such as an Si 111 double-crystal monochromator (*i.e.* 21.3 µrad Darwin width for 12.4 keV X-rays, corresponding to 1.34 × 10^−4^ bandwidth and practically constant with energy). Under this condition, the effective energy bandwidth of the outgoing beam through the monochromator depends only on the acceptance angle of the monochromator, independent of the angular divergence of the incoming beam. Therefore, to provide a high-flux beam for diffraction or scattering experiments, one can widen the effective bandwidth by simply changing the acceptance angle of the monochromator, while the angular divergence remains unchanged.

A middle energy-bandwidth monochromator covering the 10^−4^ to 10^−3^ range has the potential to contribute to a wide variety of synchrotron science demanding a higher photon flux, such as surface diffraction, small-angle scattering, time-resolved diffraction, nano-beam diffraction, coherent scattering and protein structure analysis. Indeed, in many surface diffraction experiments focusing on a few atomic layers with less than 10^−11^ reflectivity, the estimated resolution (Δ*q*) of 10^−2^ nm^−1^ in reciprocal space from a 10^−3^ bandwidth beam is sufficient for quantitative analysis. On the other hand, the wide energy-bandwidth monochromators studied so far [*e.g.* beryllium mosaic crystals (Als-Nielsen & Freund, 1992 ▸) that are low-absorption and high-reflectivity materials, or multilayer optics (Kazimirov *et al.*, 2006 ▸; Oberta *et al.*, 2012 ▸)] need further development to attain bandwidths finer than 10^−3^ (0.1%). There is great demand for reliable materials for middle energy-bandwidth monochromators to bridge the gap in the range from 10^−4^ to 10^−3^.

To meet this requirement, we revisited an asymmetric crystal that had been cut so that its surface became nonparallel with respect to the reflecting plane. To increase the photon flux available from a synchrotron X-ray source, asymmetric crystal monochromators using dynamical diffraction effects were considered (Nave, Clark *et al.*, 1995 ▸; Nave, Gonzalez *et al.*, 1995 ▸) and partly used in fixed-energy configurations (Bernstorff *et al.*, 1998 ▸). This type of configuration was proposed even in the early days of synchrotron X-ray use (Kohra *et al.*, 1978 ▸).

We adapted an asymmetrically cut Si 111 double-crystal monochromator covering the 10^−4^ to 10^−3^ bandwidths as the first optical element at the SPring-8 third-generation synchrotron facility to produce a high-flux beam optimized for surface diffraction. A narrow-divergence undulator source enabled us to tailor high-flux monochromators suitable for diffraction or scattering experiments by simply selecting the cutting angles (energy bandwidths) for asymmetric crystals. Flux gains several times higher than the standard setup were obtained from 8 to 25 keV.

## 2. Asymmetric crystals   

In dynamical X-ray diffraction, the angular acceptance of a monochromator crystal is enlarged in an asymmetric geometry characterized by the asymmetry factor *b* (Kohra, 1962 ▸); see Appendix *A* [App appa] for its definition and related parameters. Since the low-emittance undulator source at SPring-8 produces a very narrow angle-divergent but broad energy-bandwidth beam, X-ray photons in a particular energy domain are partly excluded through a monochromator. We were inspired to use these off-cast photons for an intrinsically photon-hungry experiment.

**Figure 1:**
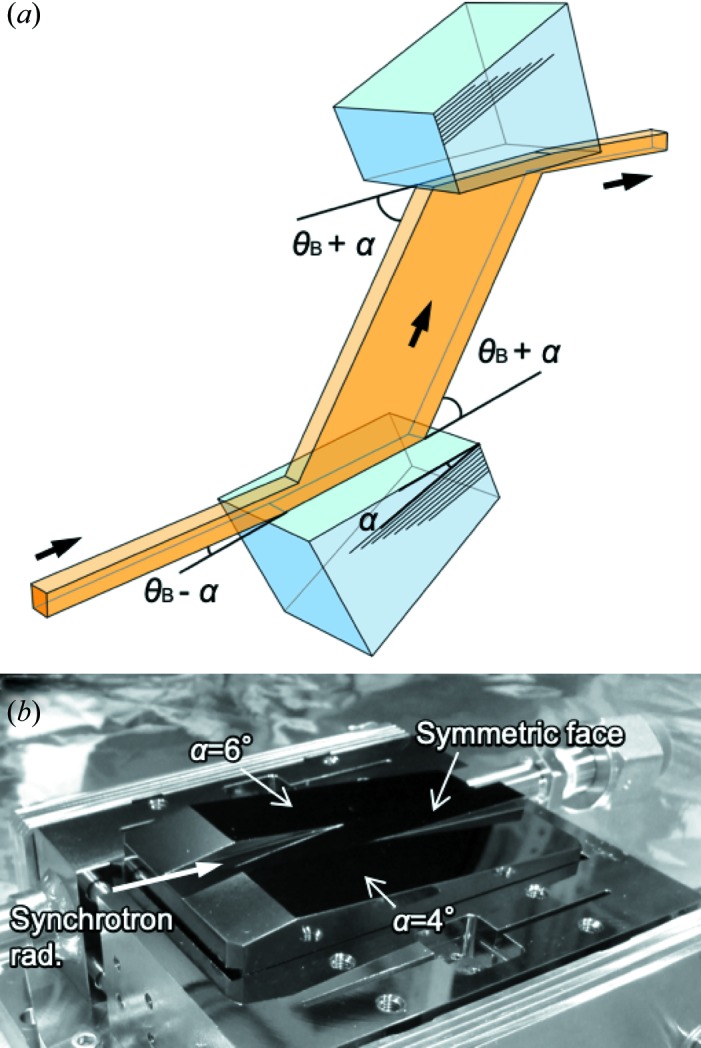
(*a*) The geometry of an asymmetrically cut Si 111 double-crystal monochromator, where θ_B_ and α denote the Bragg and asymmetry angles, respectively. (*b*) A photograph of the monolithic Si 111 crystal on the liquid-nitrogen cooled holder.

Employing an asymmetrically cut double-crystal monochromator, as shown in Fig. 1 ▸(*a*), while the beam size reflected from the first asymmetric crystal expands, the exit beam of the monochromator is compressed to the original beam size by setting the second crystal in an entirely opposite configuration to the first one. Accordingly, we are able to offer a high-flux beam while maintaining the same beam size.

**Figure 2:**
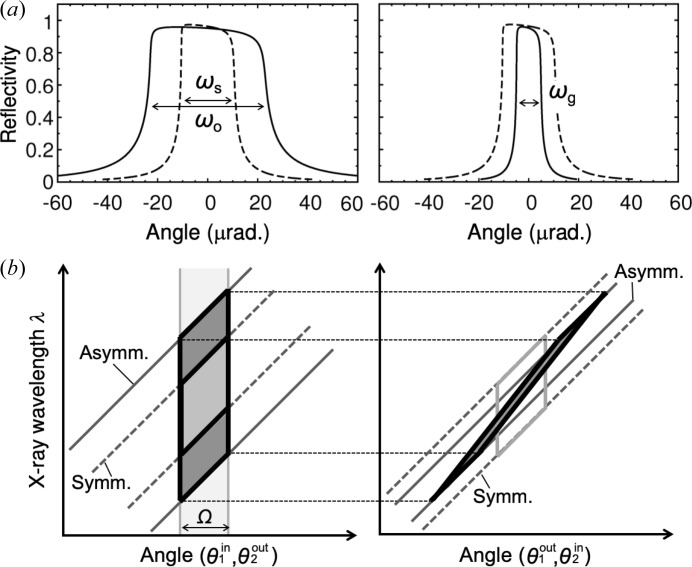
(*a*) Rocking curves in (left) the incident condition and (right) the exit condition for the first crystal at 12.4 keV with absorption. α = 6° and *b* = 0.21 in equation (3), where ω_s_, ω_o_ and ω_g_ represent the intrinsic Darwin width in the symmetric case (α = 0°), the incident acceptance and the exit reflectance, respectively. (*b*) DuMond diagrams in both symmetric and asymmetric geometries, where 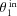 and 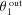 represent the incident and exit angles on the first crystal, respectively. The same applies to 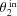 and 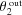 on the second crystal.

The filtering properties for an asymmetric crystal in the energy and angular domains are crucial for creating a middle energy-bandwidth monochromator for synchrotron diffraction/scattering. Calculations for the rocking curves for the Si 111 reflection in symmetric and asymmetric geometries (asymmetry angle α = 6°, the incident and exit conditions) are shown in Fig. 2 ▸(*a*). For incident and exit conditions, the diffraction width (ω) changes. Notably, towards the intrinsic Darwin width ω_s_ in the symmetric case (α = 0°), the incident acceptance ω_o_ and exit reflectance ω_g_ become broad and narrow, respectively, depending on the *b* value. The angle differences for these curves caused by refraction are omitted here. The DuMond diagrams (DuMond, 1937 ▸), graphical representations of the transfer function (TF) in diffraction events, are shown in Fig. 2 ▸(*b*). For the SPring-8 standard undulator that has a sufficiently broad energy bandwidth of *ca.* 2% to the Si 111 reflection, the source distribution becomes a vertical band with a source divergence of Ω in FWHM. As a result, the TF forms an approximate parallelogram. In the incident condition, the TF in an asymmetric geometry becomes wider than that in the symmetric one according to the wider Darwin width. Then, the TF is mapped onto the narrow and oblique area in the exit condition, where the exit angle and the wavelength are strongly correlated with each other.

At the second crystal, the opposite occurs, transferring from the narrow form to the broad one. Since the vertical angular spread of X-rays from the current SPring-8 standard undulator is narrower than the acceptance angle of the Si 111 asymmetric reflection (*e.g.* 8.2 µrad *versus* 46.2 µrad, respectively, at 12.4 keV), the outgoing beam from the double-crystal monochromator maintains its narrow angular spread as shown in Fig. 2 ▸(*b*), which is perfectly adequate for synchrotron diffraction/scattering. On the other hand, the source energy spread is much larger than the acceptance of the monochromator by at least one order of magnitude. It is a good approximation that photon flux is determined mainly by the energy acceptance of the monochromator.

The following formula can be used to calculate the photon flux *F* (photons s^−1^) through the double-crystal monochromator using the partial flux *D* [photons s^−1^ (0.1% bandwidth)^−1^] of SPring-8’s undulator light, *i.e.* a flux through an aperture:
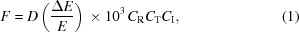
where *C*_R_ represents the effective reflectivity of the double-crystal monochromator, *C*_T_ denotes the total transmissivity of in-line materials (*i.e.* beryllium and polyimide windows, and air) and *C*_I_ is a correction term resulting from the overlap integration of the beam and the crystal acceptance in the energy domain, based on the assumption that both profiles obey a Gaussian distribution.

The energy resolution Δ*E*/*E* becomes Δ*E*/*E* = (Ω^2^ + ω^2^)^1/2^ cot θ_B_, where θ_B_ is the Bragg angle. Ω and ω are added in quadrature for expedience. Note that in the calculation of equation (1), the effective Δ*E*/*E* becomes not (Ω^2^ + ω^2^)^1/2^ cot θ_B_ but
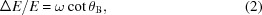
which is proportional to the area of the parallelogram in the DuMond diagram in Fig. 2 ▸(*b*).

**Figure 3:**
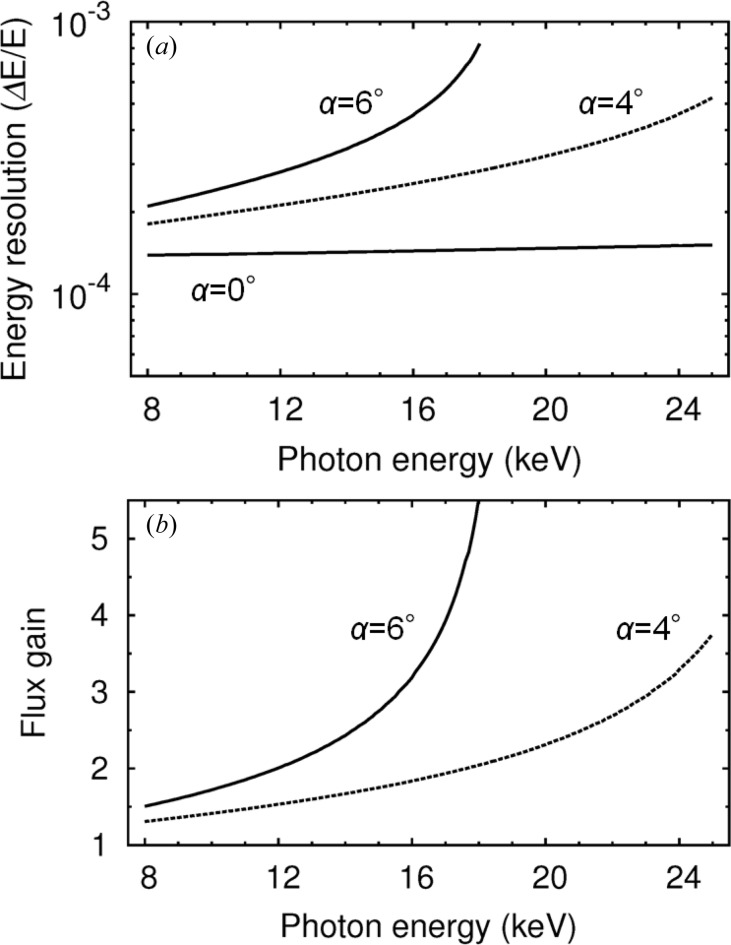
(*a*) Calculated energy resolutions for symmetric Si 111 (α = 0°) and asymmetric Si 111 (α = 6° and 4°) crystals with the SPring-8 standard undulator source, using Δ*E*/*E* = (Ω^2^ + ω^2^)^1/2^ cot θ_B_. (*b*) Calculated flux gains of asymmetrically cut double-crystal monochromators with absorption.

Fig. 3 ▸(*a*) shows calculated energy resolutions for three configurations (α = 0°, 4° and 6°). Note that the energy resolutions of the asymmetric geometries cover the middle bandwidth range from 10^−4^ to 10^−3^. In Fig. 3 ▸(*b*), the solid and dashed lines denote the flux gains for the asymmetric geometries at α = 6° and α = 4° relative to the symmetric one (α = 0°), respectively, using the effective bandwidth in equation (2), which means the flux gain becomes 1/(*b*)^1/2^. The maximum photon energies available in asymmetric geometries at α = 6° and 4° are *ca.* 18 and 25 keV, respectively, where the X-ray critical angle for total external reflection for Si gives the threshold.

This Si 111 asymmetric crystal is suitable for middle energy-bandwidth monochromators through a wide energy range from around 8 to 25 keV reaching as high as 0.1% bandwidth, as shown in Fig. 3 ▸. A major advantage of using just an asymmetric crystal is the configurability of flux gain over a wide energy range by changing only the asymmetry angle α. Both the footprint (*e.g.* with a gain of 5, expanding to 25 mm for a 1 mm size beam) and reliable materials with a proven track record such as silicon ensure an excellent heat-load capacity.

## 3. Experiments   

As shown in Fig. 1 ▸(*b*), the two Si 111 crystals for the double-crystal monochromator each have a monolithic structure with three offset surfaces consisting of two asymmetric faces at α = 4° and 6°. The symmetric face with a 10 mm width is located in the middle. The dimensions of each monochromator Si crystal are 90 × 50 × 44 mm (length in the scattering plane × width perpendicular to the plane × height). We set the asymmetry factor for the second crystal to be 1/*b*, compared with *b* for the first one in equation (3). A floating-zone synthesized Si〈111〉 ingot (ShinEtsu Ltd, Japan) was used. Cutting and mechano-chemical polishing of the surfaces were done by Sarton Works Ltd, Japan. Because the surface finish of the crystals was done by hand, small scratches slightly degrade its X-ray reflectivity in the trenches with a millimetre-scale variation of a few percent, but this is easily avoidable. By processing only crystals, no additional modifications to the liquid-nitrogen cryogenic monochromator (Yabashi *et al.*, 1999 ▸; Mochizuki *et al.*, 2001 ▸) are required. The monolithic structure using the same reflection plane offers fast switching of the reflection faces just by linear translation of the crystals, maintaining the heat-load capacity.

Measurements were made on SPring-8’s standard undulator beamline BL13XU (Sakata *et al.*, 2003 ▸), which is dedicated to revealing the structures of surface layers on solids and thin films at an atomic scale using X-ray diffraction and scattering. In both symmetric and asymmetric geometries with the Si 111 reflection, an *XY* slit for rejecting unwelcome radiation positioned 30 m after the undulator source was set to be open vertically 0.8 mm and horizontally 1.0 mm. The double-crystal monochromator was located 50 m downstream from the source. At a distance of 75 m from the source, an Si PIN photodiode (Hamamatsu Photonics Ltd, Japan, S3590-09, with a depletion layer thickness of 300 µm) was used as a detector to count the photons passing through the monochromator. From the *XY* slit to the detector, we had in-line materials, *i.e.* three beryllium windows, two polyimide films and air paths (totalling 0.75, 0.2 and 135 mm in thickness, respectively). Graphite filters and two mirrors before the *XY* slits and after the monochromator, respectively, were not used. The incident angles on the first crystal were sufficiently larger than the X-ray critical angle.

## 4. Results   

**Figure 4:**
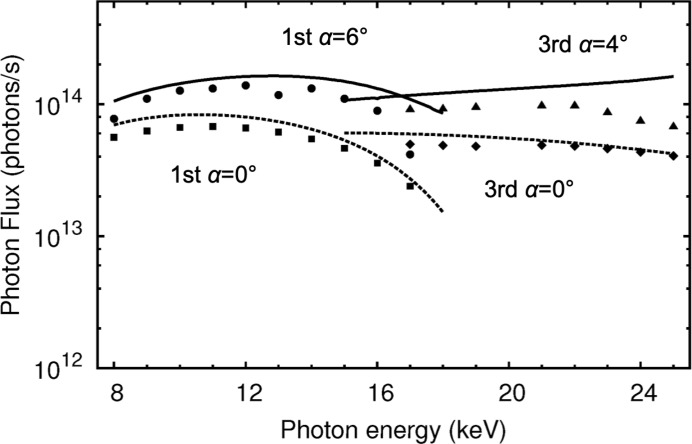
Measured photon fluxes and calculations in both symmetric and asymmetric geometries at BL13XU, SPring-8. Filled squares and diamonds represent the fluxes observed for the fundamental beam and the third harmonics, respectively, of the undulator source through the monochromator in the symmetric geometry, while filled circles and triangles represent the fluxes in asymmetric geometries at α = 6° for the fundamental beam and α = 4° for the third harmonics, respectively. The calculated photon fluxes are shown as dashed lines for the symmetric geometry and as solid lines for the asymmetric geometries.

Fig. 4 ▸ shows plots for the photon flux measured on the BL13XU beamline using the double-crystal monochromator with monolithic Si 111 crystals, and calculations in both conventional symmetric and asymmetric geometries are plotted. The contributions of higher harmonics to the fluxes were eliminated by least-squares fits of the rocking scans. The estimated statistical errors were smaller than the plot symbols in this beamline study. In the calculations of photon fluxes based on equation (1) considering the monochromator’s bandwidths in the asymmetric and symmetric geometries, the effective bandwidth in equation (2) was used. The source’s partial flux was calculated using *SPECTRA9.0* (Tanaka & Kitamura, 2001 ▸) using the following parameters: the natural emittance of an 8 GeV electron beam was set to be 3.49 nm rad; the electron beam sizes and divergences were 6.25 µm and 1.11 µrad, respectively, in the vertical direction, and 303.3 µm and 12.43 µrad, respectively, in the horizontal direction; and the total length of the undulator was 4.5 m, consisting of 140 periods of 3.2 cm periodic length. In the calculation, the typical energy spread of photons from the undulator was 1.8% at 12.4 keV with a *K* value of 1.03.

The partial fluxes of the conventional symmetric geometry through the monochromator were explained well by the calculations for both the fundamental and third harmonics of the undulator source, as shown in Fig. 4 ▸. The average error between the observations and the calculations was *ca.* 14%, where the differences probably result from unexpected absorptions at low energies. In the asymmetric geometry at α = 6°, the observed fluxes became greater than 10^14^ photons s^−1^ in the wide energy range. Currently, SPring-8 has a beam available with a lower emittance than the beam used in the current measurements. Its photon flux is 5% higher than that in Fig. 4 ▸. Nevertheless, for photon-hungry experiments, the concept of value-chain optimization from the source through the optical elements to the detector, and not relying only on improvements to the source, is becoming almost essential to conduct an experiment with sufficient efficiency. This work concerns the monochromator part.

**Figure 5:**
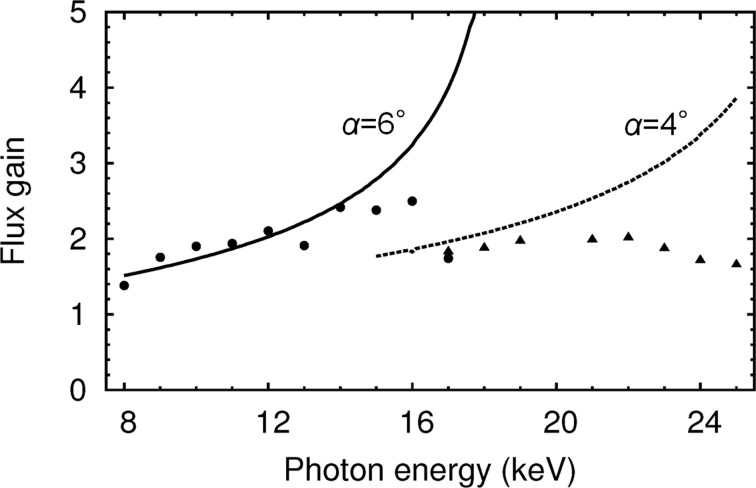
Experimental results and calculations of flux gains for asymmetric geometries compared with the conventional symmetric one. Filled circles and triangles show the observed flux gains calculated from the data in Fig. 4 ▸. Solid and dashed lines denote the same ideal curves as plotted in Fig. 3 ▸(*b*). The maximum gain obtained experimentally was 2.5.

Fig. 5 ▸ shows flux gains for the asymmetric geometries versus the symmetric one. The maximum gain obtained experimentally was 2.5. Note that the expected flux gains were obtained at lower energies, *i.e.* below 15 keV for the fundamental beam and below 20 keV for the third harmonics, while at the higher energy levels the results do not agree with expectations. On the other hand, the beam’s cross section (or foot print) on the crystal surface, which is derived by the relation in Appendix *A* [App appa], is sufficiently smaller than the crystal size (90 mm), *e.g.* 50 mm for a 2 mm size beam at the maximum gain of 5 shown in Fig. 5 ▸. The surface area accepts all the footprint. Thus, these higher-energy behaviours are explained by vibrations of the instruments caused mainly by liquid-nitrogen flow. Since ω_g_ of the first crystal and ω_o_ of the second one in equation (4) become narrower in the higher-gain region, the matching condition between the two crystals becomes severe, and then becomes sensitive to the vibrations. In the case of 17 keV at α = 6°, ω_g_ of the first crystal became only 3.6 µrad. In fact, we observed intensity fluctuations in the photodiode signal of several tens of percent in the range 50–100 Hz at 17 keV, while at the other energies showing very good flux gains the fluctuations are below the detectable level. This is because the vibrational amplitude of our monochromator on BL13XU was 0.73 µrad in terms of the misalignment angle between these two cryogenically cooled crystals (Yamazaki *et al.*, 2013 ▸).

**Figure 6:**
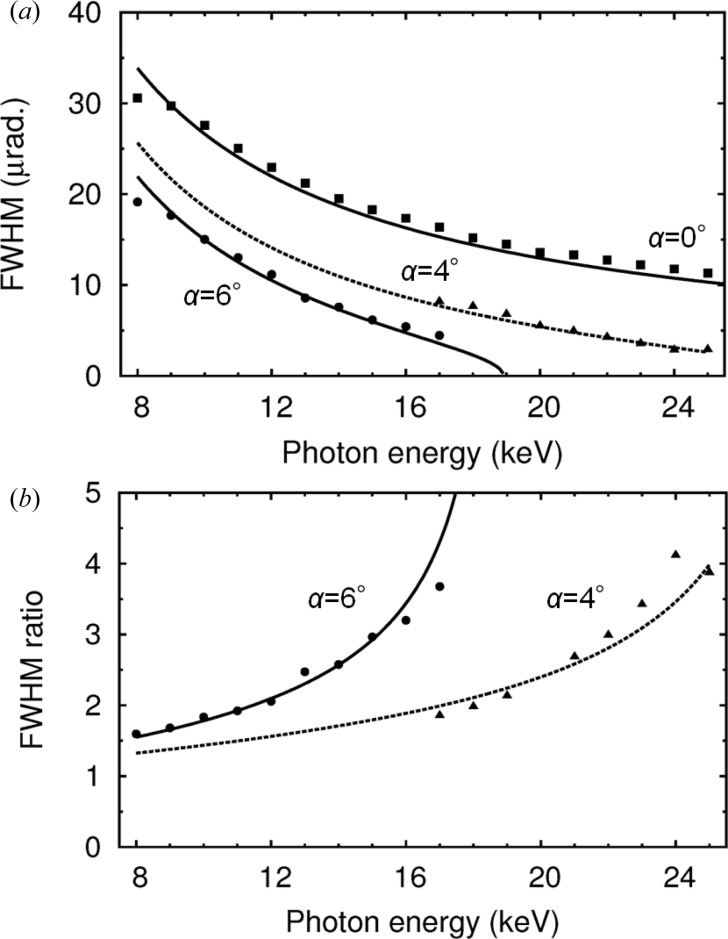
(*a*) Intrinsic FWHMs of rocking curves of the first Si 111 crystal. (*b*) FWHM ratios of asymmetric geometries compared with the symmetric one. Filled squares, circles and triangles denote the experimental results in a symmetric double-crystal geometry (α = 0°) and in asymmetric geometries at α = 6° and 4°, respectively. Solid and dashed lines denote calculations.

To evaluate the perfection of the crystal, we measured the exit reflectance widths of the first crystal, as shown in Fig. 6 ▸(*a*). The plotted results were derived based on the assumption that ω_o_ of the second crystal equalled ω_g_ of the first crystal. These FWHMs of the rocking curves agree well with calculations based on the Darwin widths of Si 111 reflections and the asymmetry factors as denoted by the lines in the figure, which suggests that the crystals themselves have produced an acceptable performance. The evaluated FWHM ratios, identical to the flux gains, of the asymmetric geometries compared with the symmetric one are shown in Fig. 6 ▸(*b*). The calculated lines denote the same ideal curves of the flux gains as plotted in Fig. 3 ▸(*b*). These experimental results agreee well with the calculations, demonstrating the designability of the system.

**Figure 7:**
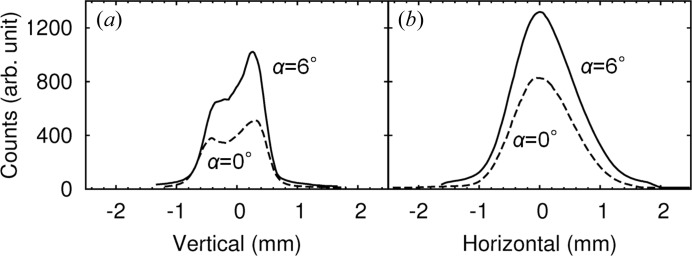
Beam profiles of 12.4 keV X-rays measured in both (*a*) vertical and (*b*) horizontal directions. Solid and dashed lines show profiles in asymmetric (α = 6°) and symmetric (α = 0°) geometries, respectively.

In addition, Fig. 7 ▸ shows beam profiles for 12.4 keV X-rays at 72 m away from the light source as measured by slit scans. The slit opening was 50 µm in the scanning direction. In both the vertical and horizontal directions, only the intensities were increased by applying the asymmetric geometry, while the beam sizes remained almost unchanged. The full widths of the beams were almost constant throughout the entire energy range from 8 to 25 keV. As mentioned in the DuMond diagram in Fig. 2 ▸(*b*), the exit beam of the asymmetric monochromator has maintained its angular divergence equivalent to the source divergence because of the conservation law. The asymmetric geometry only enhances the energy bandwidth of the beam. This property is generally suitable for diffraction and scattering experiments.

## 5. Discussion and conclusions   

In the SPring-8 upgrade plan (RIKEN & JASRI, 2012 ▸; RIKEN, 2014 ▸), the energy bandwidth for the next-generation undulator light will be *ca.* 3 × 10^−3^ as estimated from the given accelerator and undulator parameters, for which the value is still somewhat wider for direct use in many photon-hungry diffraction/scattering experiments. Furthermore, for coherent scattering, a much wider energy spread can be accepted than that provided by the symmetric Si 111 crystal monochromator typically used in synchrotron facilities (van der Veen & Pfeiffer, 2004 ▸): the symmetric monochromator provides an energy bandwidth one order of magnitude finer than the acceptable width. A middle energy-bandwidth monochromator covering the 10^−4^ to 10^−3^ range, similar to the one shown here, provides optimized beams for these experiments, and would be of use even at next-generation synchrotron sources and X-ray free-electron lasers.

The asymmetric geometry of the silicon crystals adapted in the current study is broadly applicable for this bandwidth range because of its flexible designability, pragmatic heat-load tolerance and cost effectiveness. Monochromators employing asymmetric crystals offer properties beneficial to synchrotron diffraction/scattering experiments, which generally require more flux than ever, *e.g.* for coherent, nano-beam and time-resolved diffraction/scattering applications to study dilute systems including surfaces. Indeed, on SPring-8’s current BL13XU, combinations of the asymmetric middle energy-bandwidth monochromator and focusing elements such as mirrors and refractive lenses can provide an available X-ray flux in surface diffraction that is one order of magnitude higher than the former setting, reaching 10^14^ photons s^−1^.

In conclusion, we have confirmed the effectiveness of applying asymmetric double-crystals to monochromatize narrow-divergence light from a third-generation synchrotron X-ray undulator source. The outgoing beam from an asymmetric double-crystal monochromator maintains its narrow angular spread with a low-emittance source. Moreover, a high-flux monochromator covering the 10^−4^ to 10^−3^ middle energy-bandwidths can be achieved by simply selecting the cutting angles of an asymmetric crystal. Both these features are quite sufficient for synchrotron diffraction/scattering. Using the Si 111 asymmetric double-crystal monochromator, flux gains several times higher than the standard setup were obtained from 8 to 25 keV, where an X-ray flux above 10^14^ photons s^−1^ was available at around 12 keV. The maximum gain of 2.5 was obtained relative to the standard symmetric condition.

## Appendix A

Dynamical X-ray diffraction in an asymmetric geometry is characterized by the following asymmetry factor *b*  (Kohra, 1962 ▸):

where θ_B_ is the Bragg angle, α is the angle between the crystal surface and the reflection plane, called the asymmetry angle, and is chosen to be greater than zero, and *b* < 1 in the Bragg case as shown in Fig. 1 ▸. At the incident and exit conditions, the diffraction width ω changes as follows:

where ω_s_, ω_o_ and ω_g_ represent the intrinsic Darwin width in the symmetric case (α = 0°), the incident acceptance and the exit reflectance, respectively. Similarly, the beam cross section *S* changes (*S*_g_ = *S*_o_/*b*). The asymmetry factor *b* corresponds to the intensity gain through ω_o_.
